# A survey of *Hersilia* spiders (Araneae, Hersiliidae) from Xishuangbanna, Yunnan Province, China

**DOI:** 10.3897/BDJ.12.e142805

**Published:** 2024-12-30

**Authors:** Hao Yu, Chengwen Zhang, Qianle Lu, Yejie Lin

**Affiliations:** 1 The State Key Laboratory of Southwest Karst Mountain Biodiversity Conservation of Forestry Administration, School of Life Sciences, Guizhou Normal University, Guiyang, China The State Key Laboratory of Southwest Karst Mountain Biodiversity Conservation of Forestry Administration, School of Life Sciences, Guizhou Normal University Guiyang China; 2 College of Life Sciences and Oceanography, Shenzhen University, Shenzhen, China College of Life Sciences and Oceanography, Shenzhen University Shenzhen China; 3 Institute of Zoology, Chinese Academy of Sciences, Beijing, China Institute of Zoology, Chinese Academy of Sciences Beijing China

**Keywords:** biodiversity, fauna, new record, long-spinnered spiders, taxonomy

## Abstract

**Background:**

*Hersilia* Audouin, 1826 is the largest genus of the Hersiliidae Thorell, 1869, currently including 80 extant species that are widespread throughout most of forests of the Tropical realm. The tropical rainforest in Xishuangbanna is one of the most biodiversity-rich regions in China. However, *Hersilia* can be regarded as being poorly represented in Xishuangbanna, with only two recorded species so far.

**New information:**

A survey was undertaken to study the hersiliids in Xishuangbanna. A total of four species are here addressed, raising from two to four the number of species of the genus *Hersilia* known to the area: *H.striata* Wang & Yin, 1985 and *H.lelabah* Rheims & Brescovit, 2004 that were recorded previously and *H.asiatica* Song & Zheng, 1982 (as well as the new record from Yunnan) and *H.sumatrana* (Thorell, 1890) that are recorded for the first time. *Hersilialelabah* is re-described, based on new materials and the male is described and illustrated for the first time. Detailed morphological descriptions and illustrations for both sexes of *H.lelabah* are provided. The distribution map of these four species in Xishuangbanna is given.

## Introduction

The long-spinnered spider family Hersiliidae Thorell, 1869 is a small-sized taxon, with 16 genera and 187 valid species distributed in the Old World and Australia, of which three genera and 16 species are recorded from China (WSC 2024). *Hersilia* Audouin, 1826 is the type genus of the Hersiliidae and currently includes 80 extant species that are found from the Afrotropical, Oriental and Australasian realms, comprising 43% of the total number of species of the family (WSC 2024). Despite its high species diversity, the genus *Hersilia* remains inadequately studied: nearly 1/3 of the species are known from a single sex (13 from males only, 13 from females only) (WSC 2024); types of some species might have been lost or destroyed or are difficult to locate or access (WSC 2024).

China's zoogeographical divisions are renowned for spanning both the Palearctic and Oriental realms (Gao et al. 2017). Despite *Hersilia* representing a substantial fraction of spider fauna in Tropical realm, the genus can be regarded as being poorly represented in China for a long time, with only 10 species recorded before 2020 (Zhong and Chen 2020). In the past two years, three new species and two new records have been described and reported from China and a new synonym was proposed (Lin and Li 2022, Wen et al. 2022). With that, the total number of *Hersilia* species in China reaches 14, making China the country with the most *Hersilia* species (WSC 2024). Almost all of Chinese *Hersilia* species have been described in detail, alongside high-quality illustra-tions, to allow easy species recognition (Baehr and Baehr 1993, Rheims and Brescovit 2004, Chen 2007, Dankittipakul and Singtripop 2011, Zhong and Chen 2020, Lin and Li 2022, Wen et al. 2022).

Xishuangbanna is one of the richest regions in biodiversity in China (Myers 1988). The rainforest of Xishuangbanna belongs to the transitional zone from tropical southern Asia to subtropical East Asia, containing more species diversity than similar forests in Southeast Asia (Zhu et al. 2006). Implementing an “All Species Inventory” of spiders in Xishuangbanna Tropical Botanical Garden (XTBG, 1125-hectare area in total) has increased the spider species from fewer than 50 before 2006 to about 782 by the end of 2021 (Li 2021). It is well known that *Hersilia* is frequently found in the tropical rainforests in Southeast Asia (Baehr and Baehr 1993, Rheims and Brescovit 2004, Chen 2007, Dankittipakul and Singtripop 2011). However, *Hersilia* species from Xishuangbanna are poorly studied, with only two species having been recorded: *H.striata* Wang & Yin, 1985 and *H.lelabah* Rheims & Brescovit, 2004). (Wang and Yin 1985, Lin and Li 2022).

**Figure 1.**
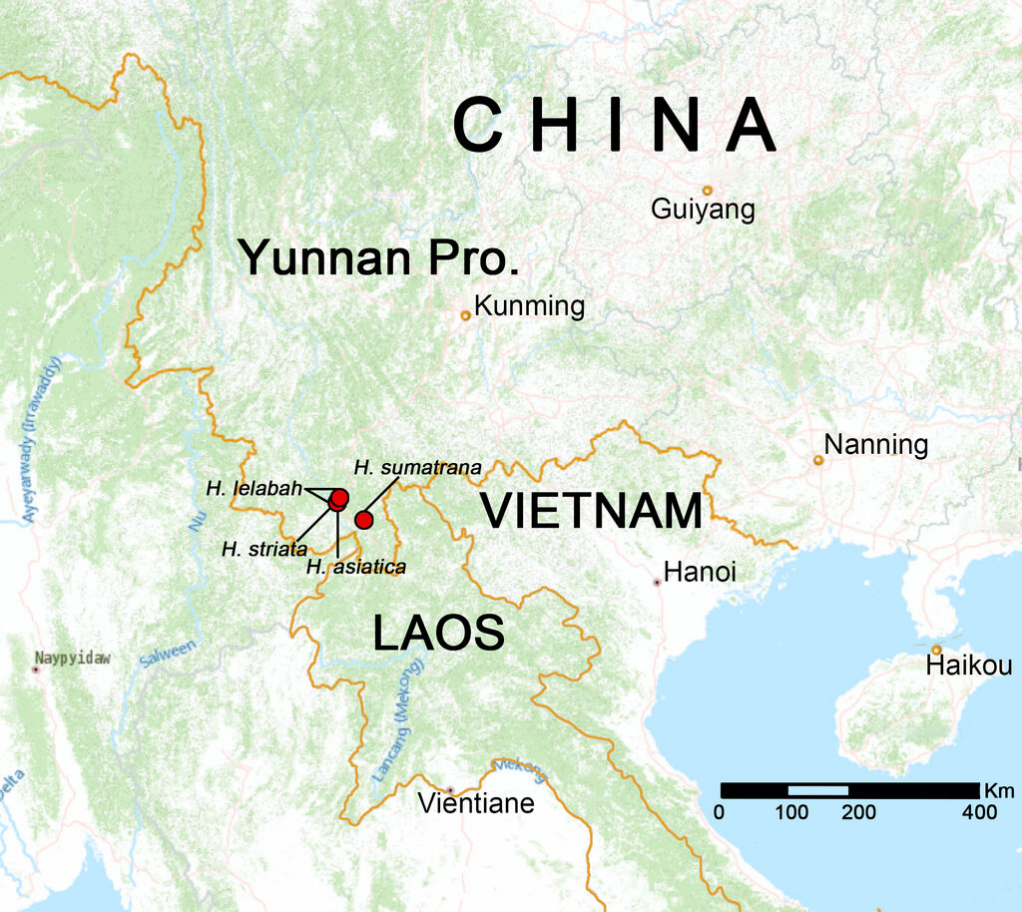
Distribution records of *Hersilia* species in Xishuangbanna.

In this paper, we undertook a survey of *Hersilia* spiders in Xishuangbanna and report four *Hersilia* species (Fig. 1), including *H.striata*, *H.lelabah* and the other two species that are recorded from Xishuangbanna for the first time: *H.asiatica* Song & Zheng, 1982 (as well as the first recorded species from Yunnan Province) and *H.sumatrana* (Thorell, 1890). The male of *H.lelabah* is described for the first time.

## Materials and methods

Specimens were primarily collected by hand-picking on tree trunks. Most of the examined materials are deposited in The State Key Laboratory of Southwest Karst Mountain Biodiversity Conservation of Forestry Administration, Guizhou Normal University (**GZNU**) in Guiyang, China, while a few others are deposited in the Institute of Zoology, Chinese Academy of Sciences (**CAS**) in Beijing, China.

Specimens were examined with an Olympus SZX7 stereomicroscope; details were studied with an Olympus BX41 compound microscope. Male palps and female epigynes were examined and illustrated after being dissected. Epigynes were removed and cleared in warm lactic acid before illustration. The vulva was also imaged after being embedded in Arabic gum. Photos were made with a Cannon EOS70D digital camera mounted on an Olympus CX41 compound microscope. The digital images were taken and assembled using Helifocus 3.10.3. software package (Khmelik et al. 2005). The distribution map was generated with ArcGis ver.10.5 (ESRI Inc 2022).

All measurements were obtained using an Olympus SZX7 stereomicroscope and given in millimetres. Eye diameters are taken at widest point. The total body length does not include chelicerae or spinnerets length. Leg lengths are given as total length (femur, patella+tibia, metatarsus, tarsus). References to figures in the cited papers are listed in lowercase (fig. or figs.); figures from this paper are noted with an initial capital (Fig. or Figs.). The terminology used in text and figure legends follows Rheims and Brescovit (2004), Foord and Dippenaar-Schoeman (2006) and Lin and Li (2022).

## Taxon treatments

### Hersilia asiatica

Song & Zheng, 1982

95278F28-77BA-558E-BBBE-423B1B1CB182

#### Materials

- **Type status:** Other material. **Occurrence:** recordedBy: Qianle Lu; individualCount: 2; sex: 1 male, 1 female; lifeStage: adult; occurrenceID: B5D33186-7D99-5990-B357-D3A22F23CA35; **Taxon:** scientificName: *Hersiliaasiatica*; order: Araneae; family: Hersiliidae; genus: Hersilia; specificEpithet: *asiatica*; taxonRank: species; scientificNameAuthorship: Song & Zheng; taxonomicStatus: accepted; **Location:** continent: Asia; country: China; countryCode: CHN; stateProvince: Yunnan; municipality: Jinghong; locality: Mengyang Town, Seasonal Rainforest; verbatimElevation: 791 m; verbatimCoordinates: 22.163056°N, 100.876972°E; decimalLatitude: 22.163056; decimalLongitude: 100.876972; georeferenceProtocol: label; **Identification:** identifiedBy: Yejie Lin; dateIdentified: 15-05-2024; **Event:** samplingProtocol: hand picking; samplingEffort: 10 km by foot; eventDate: 18/07/2022; **Record Level:** language: en; basisOfRecord: PreservedSpecimen

#### Description

**Figure 2.**
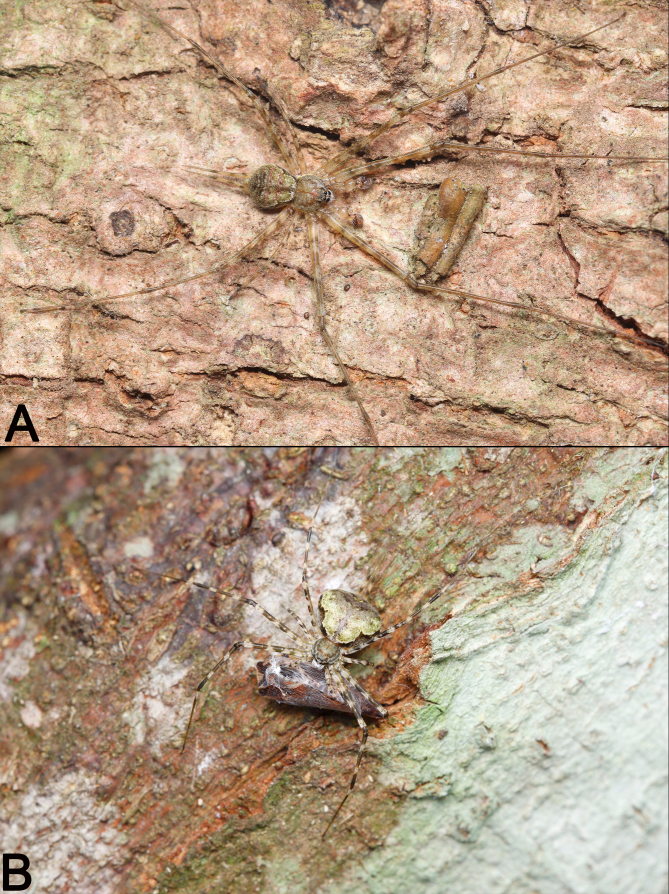
Living specimens of *Hersiliaasiatica* from Xishuangbanna. **A** Male, dorsal view; **B** Female, dorsal view.

See Zhong and Chen (2020). Live specimens as in Fig. 2A and B.

#### Distribution

China (Hunan, Guizhou, Zhejiang, Guangdong, Taiwan, Chongqing, Jiangxi, Yunnan; distribution record in Xishuangbanna as in Fig. 1), Thailand, Laos.

### Hersilia lelabah

Rheims & Brescovit, 2004

0F810B66-E0E7-56E0-B523-9CA4E45D5415

#### Materials

- **Type status:** Other material. **Occurrence:** recordedBy: Qianle Lu; individualCount: 4; sex: 1 male, 3 females; lifeStage: adult; occurrenceID: A6BF900B-7979-5AF4-8E88-FDBBA370AE84; **Taxon:** scientificName: *Hersilialelabah*; order: Araneae; family: Hersiliidae; genus: Hersilia; specificEpithet: *lelabah*; taxonRank: species; scientificNameAuthorship: Rheims & Brescovit; taxonomicStatus: accepted; **Location:** continent: Asia; country: China; countryCode: CHN; stateProvince: Yunnan; municipality: Jinghong; locality: Mengyang Town, Seasonal Rainforest; verbatimElevation: 791 m; verbatimCoordinates: 22.163056°N, 100.876972°E; decimalLatitude: 22.163056; decimalLongitude: 100.876972; georeferenceProtocol: label; **Identification:** identifiedBy: Yejie Lin; dateIdentified: 15-05-2024; **Event:** samplingProtocol: hand picking; samplingEffort: 10 km by foot; eventDate: 18/07/2022; **Record Level:** language: en; basisOfRecord: PreservedSpecimen
- **Type status:** Other material. **Occurrence:** recordedBy: Qingyuan Zhao and Zhigang Chen; individualCount: 3; sex: 3 females; lifeStage: adult; occurrenceID: 6312BB17-8CD0-581C-AA60-A01C324BE730; **Taxon:** scientificName: *Hersilialelabah*; order: Araneae; family: Hersiliidae; genus: Hersilia; specificEpithet: *lelabah*; taxonRank: species; scientificNameAuthorship: Rheims & Brescovit; taxonomicStatus: accepted; **Location:** continent: Asia; country: China; countryCode: CHN; stateProvince: Yunnan; municipality: Jinghong; locality: Guanping County, Shiwudui, Seasonal Rainforest; verbatimElevation: 888 m; verbatimCoordinates: 22.2280°N, 100.8894°E; decimalLatitude: 22.228; decimalLongitude: 100.8894; georeferenceProtocol: label; **Identification:** identifiedBy: Yejie Lin; dateIdentified: 01-01-2022; **Event:** samplingProtocol: hand picking; samplingEffort: 10 km by foot; eventDate: 20/07/2012; **Record Level:** language: en; basisOfRecord: PreservedSpecimen

#### Description

**Figure 3.**
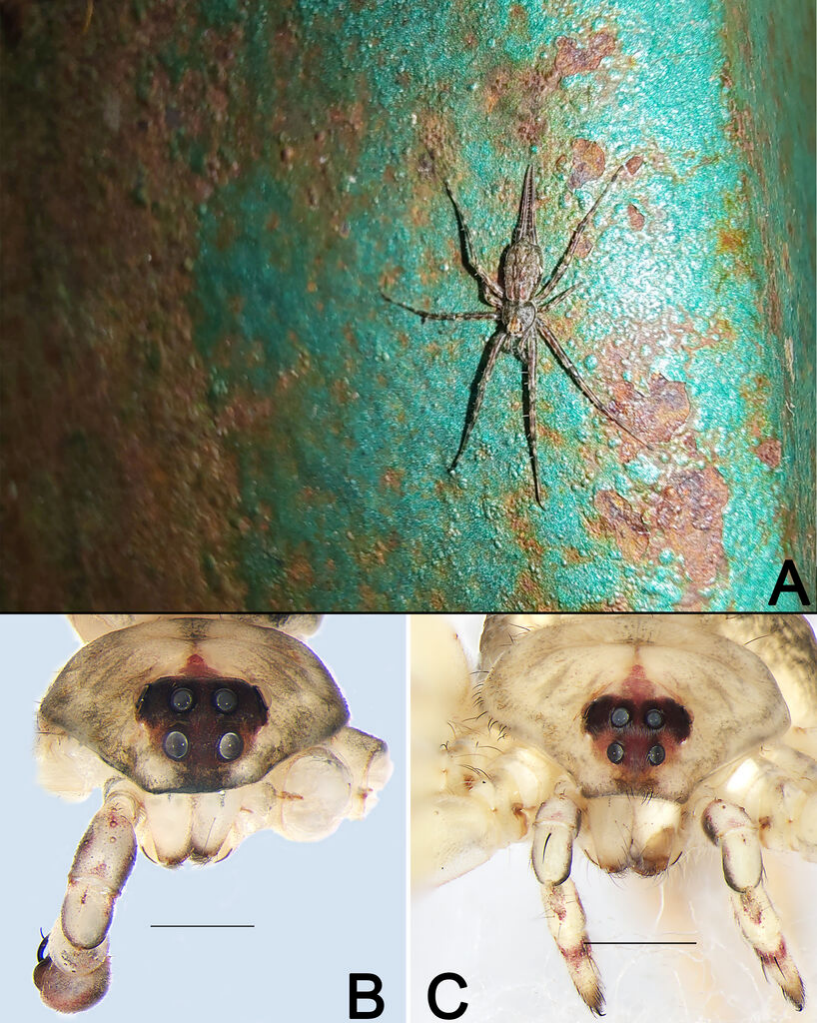
*Hersilialelabah* from Xishuangbanna, living specimen (**A**) and frontal views of prosoma (**B, C**). **A** Female, dorsal view; **B** Male; **C** Female. Scale bars: 0.5 (**B**), 1 mm (**C**).

**Figure 4.**
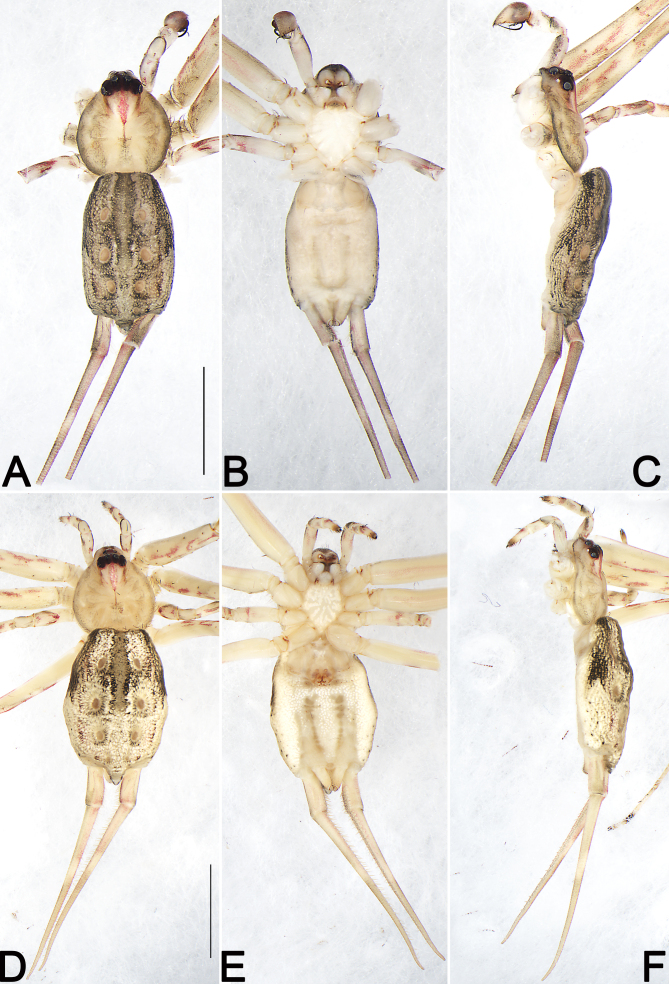
Habitus of *Hersilialelabah*, male (**A–C**) and female (**D–F**). **A, D** Dorsal view; **B, E** Ventral view; **C, F** Lateral view. Scale bars: 2 mm (equal for **A–C**), 3 mm (equal for **D–F**).

***Male*** (Fig. 4A–C and Fig. 3B). Total length 4.69; carapace 1.91 long, 1.76 wide; abdomen 2.78 long, 1.77 wide.

*Carapace* pear-shaped, basically yellowish-white with border black, with a short and red, sword-shaped medial band starting from behind PME, almost reaching black fovea; cervical groove and radial grooves distinct; eye area dark brown with black eye rings (Fig. 4A, C and Fig. 3B). In dorsal view, both anterior eye row (AER) and posterior eye row (PER) are distinctly recurved, PER slightly wider than AER (Fig. 4A). Eye sizes and interdistances: anterior median eyes (AME) 0.19, anterior lateral eyes (ALE) 0.04, posterior median eyes (PME) 0.15, posterior lateral eyes (PLE) 0.14; distance between AMEs (AME–AME) 0.19, distance between AME and ALE (AME–ALE) 0.15, distance between PMEs (PME–PME) 0.11, distance between PME and PLE (PME–PLE) 0.19. Length of median ocular quadrangle (MOQ) 0.42, MOQ anterior width 0.42, MOQ posterior width 0.39. Clypeal height 0.18. *Chelicerae* with white base and red wine-coloured fangs, with 3 teeth on promargin and 5 on retromargin (Fig. 4A–C). Sternum, labium and endites nearly cream-coloured (Fig. 4B). Sternum 1.03 long, 0.93 wide; labium 0.28 long, 0.32 wide; endite 0.49 long, 0.25 wide.

*Abdomen* dorsally brownish-cream-coloured with brown pattern; with a pair of large lateral dark bands on anterior half; cardiac impression ribbon-shaped, wide; with four pairs of orange-brown muscle impressions and the second pair largest; venter uniformly yellowish-white, without pattern. bS (basal segment of posterior lateral spinneret) 0.84, tS (basal segment of posterior lateral spinneret) damaged (Fig. 4A–C).

*Legs* uniformly light yellow, except several reddish, irregular streaks on femur, patella, tibia, metatarsus and tarsus. Leg length: I (3.54, 3.89, —, —), II 11.93 (3.39, 3.63, 4.53, 0.38), III 3.83 (1.20, 1.16, 0.97, 0.50), IV missing (Fig. 4A–C).

**Figure 5.**
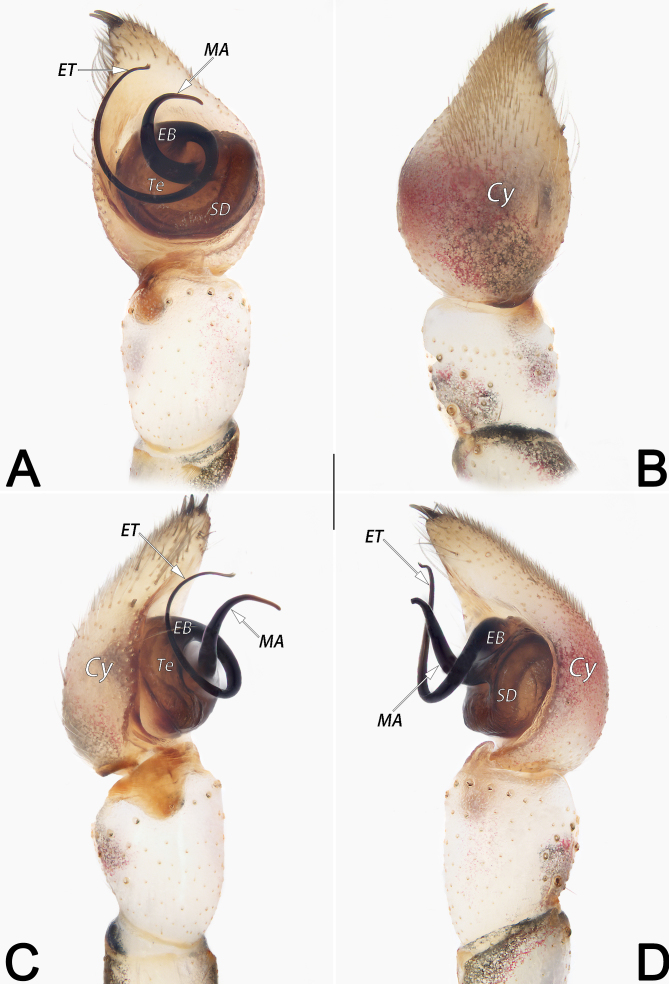
Male palp of *Hersilialelabah*. **A** Ventral view; **B** Dorsal view; **C** Prolateral view; **D** Retrolateral view. Scale bar: 0.2 mm (equal for **A–D**). Abbreviations: Cy, cymbium; EB, embolar base; ET, embolar tip; MA, median apophysis; SD, sperm duct; Te, tegulum.

*Palp* (Fig. 5A–D). Tibia long, ca. 1.3 × patella length, apically with three strong spines. Cymbium (Cy) long, ca. 1.55× longer than wide, distally armed with 3 strong apical spines. Tegulum (Te) transversely elongated, oval and slightly bulging, ca. 1.5× wider than long; sperm duct wide and distinct, orientated clockwise along the margin of the tegulum. Embolar base (EB) represented by enlarged tubercle, inserted at approximately 11–12 o’clock of tegulum, partly hidden behind median apophysis; the free part of embolus (ET) filamentous, orientated clockwise, shaped like the number 6, its tip terminating at ca. 11 o’clock position. Median apophysis (MA) C-shaped, originating at central portion of tegulum, gradually tapering towards tip, apex sharp and retrolaterally pointed, terminating at ca. 1 o’clock position.

***Female*** (Fig. 4D–F and Fig. 3C). Total length 7.21; carapace 2.71 long, 2.62 wide; abdomen 4.50 long, 3.36 wide. Eye sizes and interdistances: AME 0.14, ALE 0.08, PME 0.17, PLE 0.15; AME–AME 0.18, AME–ALE 0.21, PME–PME 0.13, PME–PLE 0.23. MOQL 0.48, MOQA 0.46, MOQP 0.47. Sternum 1.68 long, 1.29 wide. Measurements of legs: I 13.73 (4.08, 4.01, 4.60, 1.04), II 12.64 (4.27, 4.46, 3.37, 0.54), III 9.57 (2.93, 3.25, 2.25, 1.14), IV 11.02 (3.87, 3.56, 3.06, 0.53). General characters as in male, but distinctly larger in size and lighter in colour (Fig. 4D–F and Fig. 3C). See Rheims and Brescovit (2004) for others described.

**Figure 6.**
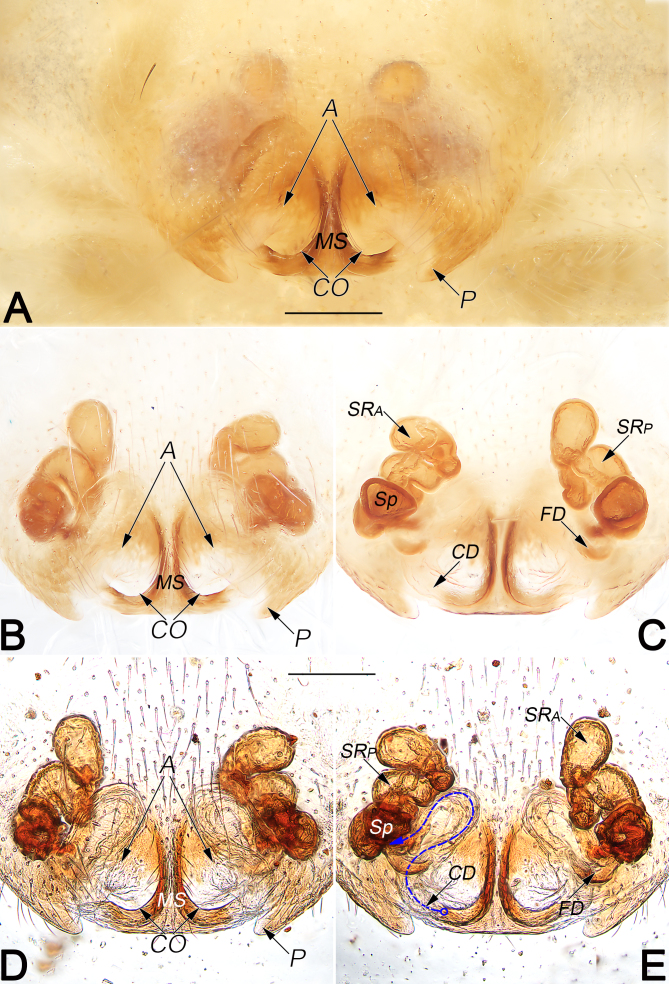
Female genitals of *Hersilialelabah*. **A** Epigyne, intact, ventral view; **B** Epigyne, cleared, ventral view; **C** Vulva, cleared, dorsal view; **D** Epigyne, cleared and embedded in Arabic gum, ventral view; **E** Vulva, cleared and embedded in Arabic gum, dorsal view. Scale bar: 0.2 mm (equal for **A–E**). Abbreviations: A, atrium; CD, copulatory duct (dashed line showing schematic course of copulatory duct, dorsal); CO, copulatory opening; FD, fertilisation duct; MS, median septum; P, pocket; Sp, spermatheca; SRA, anterior chamber of seminal receptacle; SRP, posterior chamber of seminal receptacle.

*Genitals* (Fig. 6A–E). Epigynal plate distinctly wider than long, anterior and lateral margins not rebordered; the arrangement of the various parts of the vulva are indistinctly visible through the tegument. Median septum (MS) nearly⊥-shaped, consisting of a longitudinal stem and a transverse base; septal stem distinctly narrow, about 2/5 length of epigynal plate and about 1/7 (the narrowest distance)–1/3 (the widest distance) width of septal base; septal base ca. 1/3 of epigyne width. Epigynal atrium (A) large, located at the posterior portion of epigynal plate, divided by septum, represented by two symmetrical, egg-shaped cavities. Copulatory openings (CO) indistinct, located at basal atrial borders. The two lateral borders represented by triangular pockets (P), distinctly protruding. Copulatory ducts (CD) hyaline and sinuous, almost invisible, ascending in parallel, forming two S-shaped courses, finally connecting to spermathecae. Spermathecae (Sp) small, bean-shaped, located laterally. Seminal receptacles subdivided in 2 chambers, the anterior chamber (SRA) globular and with a sinuous stalk, while the posterior chamber (SRP) tubular. Fertilisation ducts (FD) located on the posterior surface of spermathecae, curved and acicular.

#### Diagnosis

Medium-sized hersiliids, belonging to the *caudata* species-group. Males of *H.lelabah* are similar to those of *H.caudata* (type species of *Hersilia*, the core species of the *caudata*-group, see Baehr and Baehr (1993): 17, figs. 15c–d, Foord and Dippenaar-Schoeman (2006): 59, figs. 132–133) for the general shape of male palp, but can be differentiated from *H.caudata* by: (1) median apophysis C-shaped, its apex terminating at ca. 1 o’clock position, pointing retrolaterally (vs. hook-shaped, terminating at ca. 6–7 o’clock position, pointing proximally) (cf. Fig. 5A, C, D and Baehr and Baehr (1993): figs. 15c–d); (2) embolus distinctly longer, its tip terminating at ca. 11 o’clock position (vs. proportionally shorter, its tip terminating at ca. 6 o’clock position) (cf. Fig. 5A and Baehr and Baehr (1993): fig. 15c). Females of *H.lelabah* can be distinguished from all other members of the *caudata*-group by the ⊥-shaped median septum and by the seminal receptacles subdivided in 2 chambers (Fig. 6A–E).

#### Distribution

China (Yunnan; distribution record in Xishuangbanna as in Fig. 1), Malaysia.

#### Notes

*Hersilialelabah* was described, based on two female specimens from Kinabalu National Park of Sabah, Malaysia and was assigned to the *H.caudata*-group in the original paper (Rheims and Brescovit 2004) and recently reported from China for the first time by Lin and Li (2022), based on three females from Xishuangbanna. However, the male of *H.lelabah* has not been found so far (Rheims and Brescovit 2004, Lin and Li 2022, WSC 2024).

While examining the spider specimens collected from Xishuangbanna, we found pairs of *Hersilia* specimens in the same location. Therefore, it is very likely they are the opposite sexes of the same species. Based on the somatic and epigynal charaters, we identified the female as *H.lelabah* (Rheims and Brescovit 2004). The habitus, markings and leg spination of the male specimen is similar to the female *H.lelabah* (Fig. 3B, C and Fig. 4) and palpal structures conform to the *caudata*-group. As a result, we matched the females and males together as *H.lelabah*.

A difference is exhibited between the newly-collected females and the original drawing by Rheims and Brescovit (2004), related to different courses of copulatory ducts: copulatory ducts forming two S-shaped courses in the new materials, while ascending obliquely in the original drawing (cf. Fig. 6E and Rheims and Brescovit (2004): fig. 9). In consideration of that, copulatory ducts are totally transparent and almost invisible in alcohol, while the S-shaped courses of copulatory ducts can be easily overlooked (as in Fig. 6C). Despite of the difference just mentioned, our new materials bear a striking similarity to the original illustrations: the bean-shaped spermathecae and the subdivided seminal receptacles, the globular anterior chamber with a sinuous stalk, the tubular posterior chamber and the similar abdominal pattern with a ribbon-shaped cardiac impression and pairs of orange-brown muscle impressions, leave no doubts that our identification is correct.

### Hersilia striata

Wang & Yin, 1985

D216BC57-4679-5876-AB2C-BAC8D8E65D8D

#### Materials

- **Type status:** Other material. **Occurrence:** recordedBy: Qianle Lu; individualCount: 3; sex: 1 male, 2 females; lifeStage: adult; occurrenceID: 0ABA8346-3CB7-500A-BCA3-BE9984F5CCE0; **Taxon:** scientificName: *Hersiliastriata*; order: Araneae; family: Hersiliidae; genus: Hersilia; specificEpithet: *striata*; taxonRank: species; scientificNameAuthorship: Wang & Yin; taxonomicStatus: accepted; **Location:** continent: Asia; country: China; countryCode: CHN; stateProvince: Yunnan; municipality: Jinghong; locality: Mengyang Town, Seasonal Rainforest; verbatimElevation: 791 m; verbatimCoordinates: 22.163056°N, 100.876972°E; decimalLatitude: 22.163056; decimalLongitude: 100.876972; georeferenceProtocol: label; **Identification:** identifiedBy: Yejie Lin; dateIdentified: 15-05-2024; **Event:** samplingProtocol: hand picking; samplingEffort: 10 km by foot; eventDate: 81/07/2022; **Record Level:** language: en; basisOfRecord: PreservedSpecimen

#### Description

**Figure 7.**
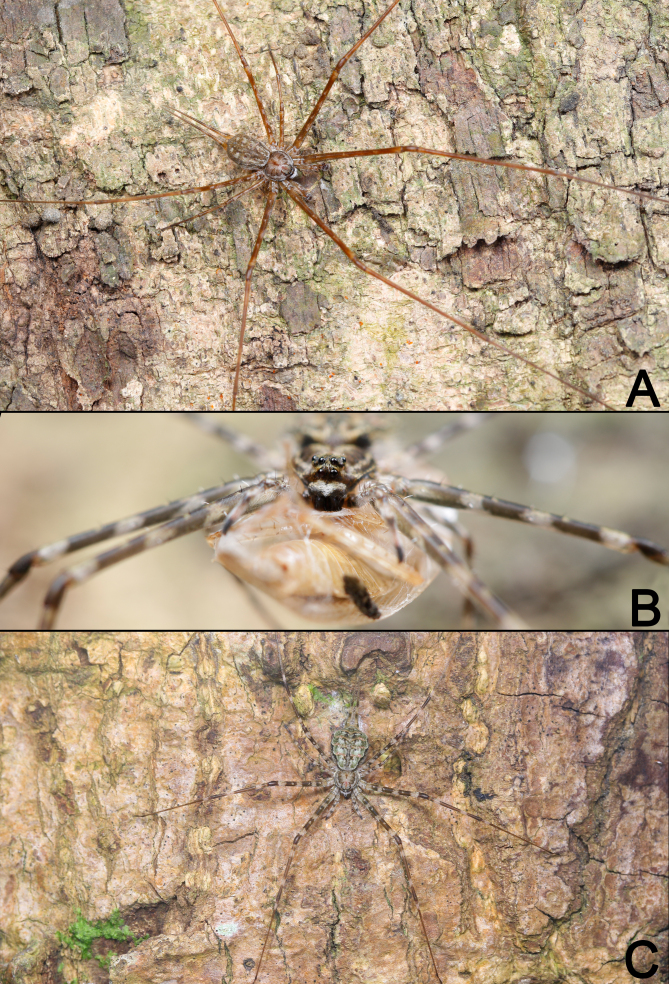
Living specimens of *Hersiliastriata* from Xishuangbanna. **A** Male, dorsal view; **B** Female, frontal view; **C** Female, dorsal view.

See Baehr and Baehr (1993). Live specimens as in Fig. 7.

#### Distribution

China (Guangdong, Taiwan, Yunnan; distribution record in Xishuangbanna as in Fig. 1), India, Myanmar, Thailand, Indonesia (Java, Sumatra).

### Hersilia sumatrana

(Thorell, 1890)

D5672D6F-2F15-57B8-ABED-B43DE3DD6C00

#### Materials

- **Type status:** Other material. **Occurrence:** recordedBy: Qianle Lu; individualCount: 2; sex: 2 females; lifeStage: adult; occurrenceID: B40B99CF-2CE7-5ABD-BABB-FF251A7411A0; **Taxon:** scientificName: *Hersiliasumatrana*; order: Araneae; family: Hersiliidae; genus: Hersilia; specificEpithet: *sumatrana*; taxonRank: species; scientificNameAuthorship: Thorell, 1890; taxonomicStatus: accepted; **Location:** continent: Asia; country: China; countryCode: CHN; stateProvince: Yunnan; county: Mengla; locality: Menglun Town, Xishuangbanna Tropical Botanic Garden (XTBG), G213 roadside; verbatimElevation: 581 m; verbatimCoordinates: 21.90666°N, 101.265°E; decimalLatitude: 21.90666; decimalLongitude: 101.265; georeferenceProtocol: label; **Identification:** identifiedBy: Yejie Lin; dateIdentified: 15-05-2024; **Event:** samplingProtocol: hand picking; samplingEffort: 10 km by foot; eventDate: 30/06/2023; **Record Level:** language: en; basisOfRecord: PreservedSpecimen

#### Description

**Figure 8.**
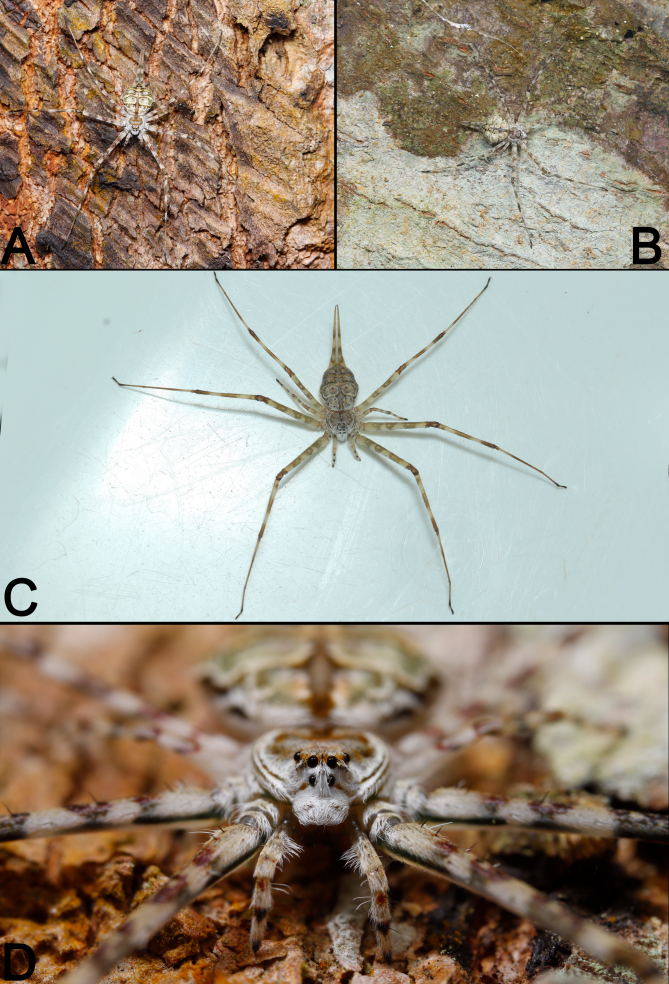
Living specimens of *Hersiliasumatrana* from Xishuangbanna, females. **A–C** Dorsal view; **D** Frontal view.

See Baehr and Baehr (1993). Live specimens as in Fig. 8.

#### Distribution

China (Yunnan; distribution record in Xishuangbanna as in Fig. 1), India, Myanmar, Malaysia, Vietnam, Philippines, Brunei, Indonesia (Sumatra, Borneo)

## Supplementary Material

XML Treatment for Hersilia asiatica

XML Treatment for Hersilia lelabah

XML Treatment for Hersilia striata

XML Treatment for Hersilia sumatrana
